# Microbial pyrazine diamine is a novel electrolyte additive that shields high-voltage LiNi_1/3_Co_1/3_Mn_1/3_O_2_ cathodes

**DOI:** 10.1038/s41598-022-22018-1

**Published:** 2022-11-25

**Authors:** Agman Gupta, Rajashekar Badam, Noriyuki Takamori, Hajime Minakawa, Shunsuke Masuo, Naoki Takaya, Noriyoshi Matsumi

**Affiliations:** 1 Graduate School of Advanced Science and Technology, Japan Advanced Institute of Science and Technology (JAIST), 1-1 Asahidai, Nomi, Ishikawa 923-1292 Japan; 2 Faculty of Life and Environmental Sciences, Microbiology Research Center for Sustainability, University of Tsukuba, 1-1-1 Tennodai, Tsukuba, Ibaraki 305-8572 Japan

**Keywords:** Microbiology, Energy science and technology, Materials science

## Abstract

The uncontrolled oxidative decomposition of electrolyte while operating at high potential (> 4.2 V vs Li/Li^+^) severely affects the performance of high-energy density transition metal oxide-based materials as cathodes in Li-ion batteries. To restrict this degradative response of electrolyte species, the need for functional molecules as electrolyte additives that can restrict the electrolytic decomposition is imminent. In this regard, bio-derived molecules are cost-effective, environment friendly, and non-toxic alternatives to their synthetic counter parts. Here, we report the application of microbially synthesized 2,5-dimethyl-3,6-bis(4-aminobenzyl)pyrazine (DMBAP) as an electrolyte additive that stabilizes high-voltage (4.5 V vs Li/Li^+^) LiNi_1/3_Mn_1/3_Co_1/3_O_2_ cathodes. The high-lying highest occupied molecular orbital of bio-additive (DMBAP) inspires its sacrificial in situ oxidative decomposition to form an organic passivation layer on the cathode surface. This restricts the excessive electrolyte decomposition to form a tailored cathode electrolyte interface to administer cyclic stability and enhance the capacity retention of the cathode.

## Introduction

The ever-increasing demand of next-generation electric vehicles (EVs), hybrid electric vehicles (HEVs), portable consumer electronics, and power grids has led to extensive research towards developing high-energy density cathodes in lithium-ion batteries (LIBs)^1–4^. As suitable alternatives to the conventional state-of-the-art LiCoO_2_ cathodes in LIBs, a variety of cathode materials based on oxides of different blends of transition metals (Ni, Mn, and Co-NMC) have been investigated as they offer higher specific capacity and operating potentials^5–9^. Among a plethora of newly investigated cathode materials, LiNi_1/3_Mn_1/3_Co_1/3_O_2_ cathode has been recognized to deliver excellent performance while operating at high potential (~ 4.5 V vs Li/Li^+^) with high reversible capacity^10–12^. Though these cathodes can deliver superior performance at a higher potential in comparison to their conventional counterparts, the excessive oxidative decomposition of the carbonate species in commercial electrolytes at high potential because of their high-lying highest occupied molecular orbital (HOMO) leads to the formation of an unstable and thick cathode electrolyte interphase (CEI) with high interfacial impedance on the cathode surface^13–17^. This affects the performance of cathodes severely with poor cyclic stability and compromised reversible capacity. Also, the decomposition of lithium salt LiPF_6_ (LiPF_6_ → LiF + PF_5_) and the presence of water in trace quantity further lead to other detrimental reactions (PF_5_ + H_2_O → PF_3_O + 2HF) resulting in HF generation that can adversely affect the integrity of CEI and corrode cathode morphology irreversibly^18–21^. Therefore, the surface of high-performance NMC-cathodes like LiNi_1/3_Mn_1/3_Co_1/3_O_2_ is highly susceptible to adverse degradative response and reactions of the electrolyte while operating at higher potential.

One of the best strategies to restrict the uncontrolled oxidative decomposition of commercial electrolyte species has been the use of functional organic molecules as electrolyte additives that can mask the surface of the cathode by forming a protection layer before the degradative response of the electrolyte species at a higher potential^22,23^. Therefore, to stabilize high-performance NMC-cathodes, a variety of additives like 1,3-propane sultone^24^, vinyl carbonate (VC)^25^, succinic anhydride^26^, (4,4´-bi(1,3,2-dioxathiolane))2,2´-dioxide (BDTD)^27^, bis(trimethylsilyl) 2-methyl-2-fluoromalonate (BTMSMFM)^28^, lithium bisoxalatodifluorophosphate (LiBODFP)^29^, etc. have been investigated. Though the application of these additives could be partially effective towards stabilizing the interaction of carbonate-based electrolytes with cathodes, the problem of maintaining the structural stability of cathode was not addressed convincingly. Therefore, other techniques like cathode materials coating with ZnO, Al_2_O_3_, etc. were stand-alone options^30,31^.

Hence, there has been a need of a versatile additive that can not only supress the oxidative decomposition of the electrolyte species by forming an armour layer on the cathode surface but also can stabilize structure of transition metal oxides on the cathode. Taking a big step towards designing a versatile electrolyte additive that can address multiple problems encountered while working with cathodes (both at the electrolyte-front and cathode-front), recently, our group reported a novel bisiminoacenaphthenequinone (BIAN)-based diamine additive (BIANODA) with a strategic framework of π-s-p (π-spacer-polyemrizable group) as ligand-inspired electrolyte additive to stabilize high energy density NMC cathodes^32^. However, the above-mentioned state-of-the art electrolyte additives are not environmentally benign as they are synthetically formulated for battery applications involving the use of hazardous, toxic, and expensive chemicals. Therefore, the synthesis and application of synthetic add-ons like electrolyte additives in LIBs not only raises safety and environmental concerns but also increases the cost of manufacturing.

**Scheme 1:**
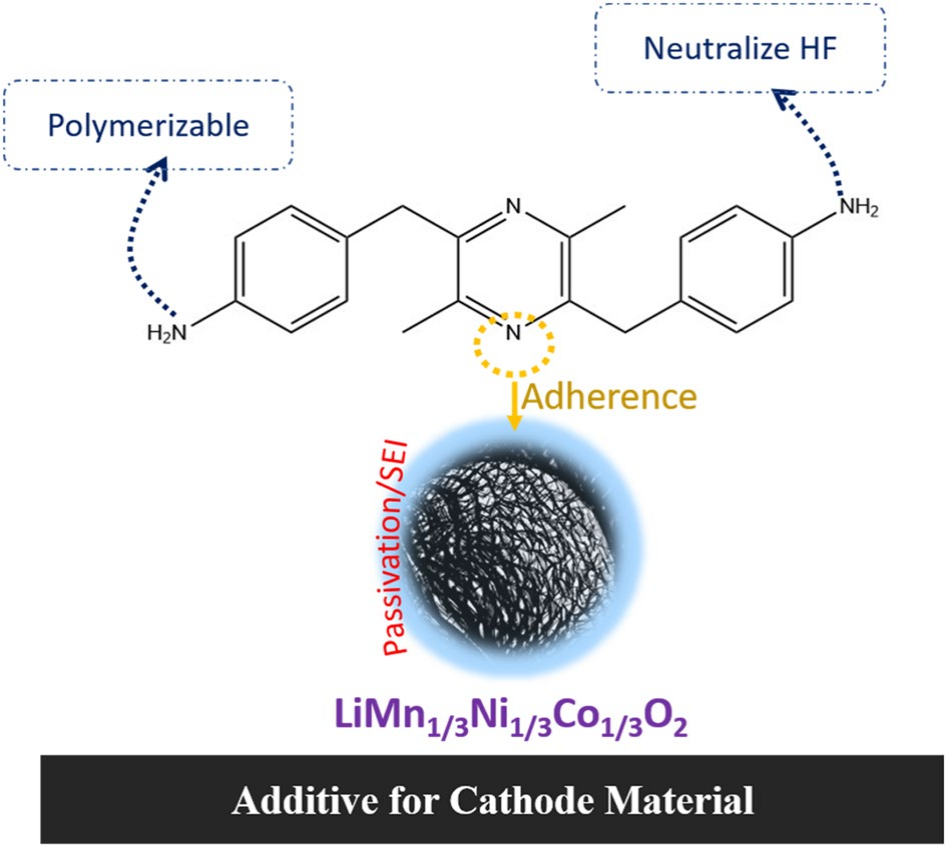
Structural significance and mode of action of DMBAP additive to stabilize LIB cathodes.

In this regard, bio-based molecules deserve necessary attention for their application to stabilize next-generation electrode materials in LIBs^33,34^. Being derived from natural sources, bio-based molecules create an avenue for decreasing the cost, sophistication, and hazards associated with complex syntheses. Also, they add a green footprint and sustainable perspective to LIB. Further, microbial synthesis enables simple one-pot fermentation reaction of complicated chemical compounds that otherwise will need many reaction steps followed by purification in ordinary synthetic chemistry. This suggests the great potential of biomaterials in energy devices to optimize the significance of sustainability and biocompatibility. For a biomolecule to mimic its synthetic counterpart in energy storage devices, an essential requirement is the presence of alike functional groups that are electrochemically relevant and operable in devices. This inspired a rigorous collaboration between biochemists and electrochemists to tread a transdisciplinary path in creating a possibility of sustainable future with energy devices running on bio-based or partially bio-based materials. As mentioned earlier, with the successful application of BIANODA as electrolyte additive to stabilize LiNMC cathode in LIBs, we realized the relevance of organic molecules with strategic framework of π-s-p (π-spacer-polyemrizable group) to act as electrolyte additives in LIBs towards stabilizing cathode materials. And one such transdisciplinary collaboration led us to choose a biomolecule that had a strong candidature as an electrolyte additive. Therefore, as a sustainable, eco-friendly, cost-effective, and non-toxic alternative, here, we report the application of a microbially synthesized 2,5-dimethyl-3,6-bis(4-aminobenzyl)pyrazine (DMBAP) as an electrolyte additive to stabilize LiNi_1/3_Mn_1/3_Co_1/3_O_2_ cathodes. We discovered the *Pseudomonas fluorescens* SBW25 gene cluster for the biosynthesis DMBAP^35^. The gene cluster encode the 4-aminophenyleamine (4APhe)-biosynthetic enzymes, and 4APhe C-acetyltransferase, dihydropyrazine oxidase. The aminoketone generated by de novo synthesized 4APhe and 4APhe C-acetyltransferase was spontaneously condensed into dihydropyrazine followed by oxidation of hydropyrazine to yield DMBAP^35^. We also developed recombinant bacteria that ferments glucose to DMBAP, which provides us the novel DMBAP of the biomass-derived natural pyrazine harbouring distinct aromatic diamine moieties. Scheme 1 depicts the significance of bio-additive DMBAP to stabilize high-voltage LiNi_1/3_Mn_1/3_Co_1/3_O_2_ cathodes. Some salient features of the DMBAP molecule that highlight its structure–property relevance to stabilize high-voltage LiNi_1/3_Mn_1/3_Co_1/3_O_2_ cathodes in a LIB are: (i) suitable highest occupied molecular orbital (HOMO) band energy that can empower it to undergo sacrificial in-situ oxidative decomposition on the cathode surface leading to the formation of a protection layer to prevent the excessive decomposition of electrolyte species. Hence, this phenomenon would potentially inspire the formation of a controlled CEI. (ii) The diamine of DMBAP can act as HF scavenger to prevent the dissolution of CEI because the in-situ generation of HF as electrolyte decomposition by-product is detrimental for the longevity of CEI for durable cyclability. (iii) Also, electron rich diimine nitrogen atoms can potentially latch onto active metal centres of the cathode to adhere the surface protection layer on the cathode surface. Therefore, the novel application of DMBAP biomolecule as an electrolyte additive in LIBs provides a wide spectrum of insights on the rational designs of bio-based molecules for next-generation LIBs as well as sustainable advancements in future energy storage devices.

## Results and discussion

### Theoretical studies

**Figure 1:**
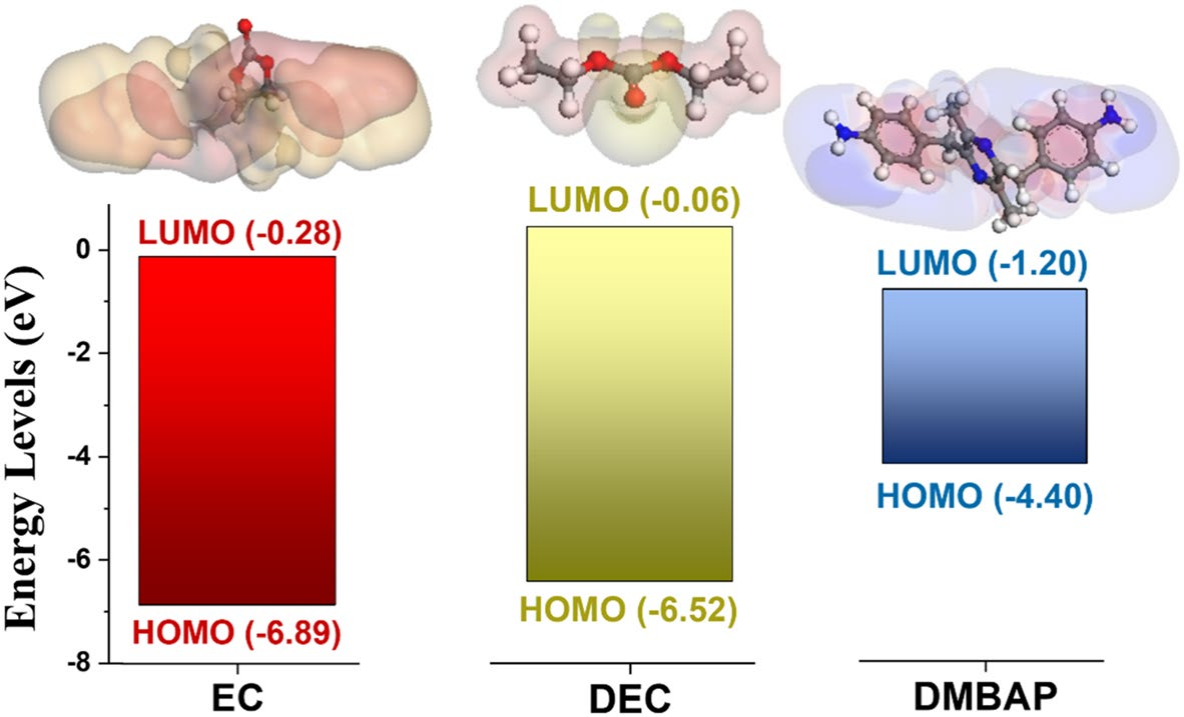
Theoretical evaluation of electrolyte components and DMBAP bio-additive. HOMO–LUMO band energy comparison between the electrolyte components ethylene carbonate (EC), diethyl carbonate (DEC), and DMBAP additive, respectively with their corresponding DFT optimized structures.

Though being theoretically stable up to 6 V versus Li/Li^+^, due to the catalytic properties of the metal centres on the cathode, most carbonate-based electrolytes undergo oxidative decomposition at lower potentials^23,36^. The reason behind their pre-mature oxidation at a lower potential is their high HOMO band energy level that makes them susceptible to oxidative decomposition while operating at high potential. Therefore, the rationale behind the design of an effective additive must consider the appropriate design of its HOMO–LUMO band energies with HOMO band energy particularly higher than that of electrolyte species to restrict the uncontrolled oxidative decomposition of the electrolyte on the cathode surface. Therefore, to understand the energy levels of DMBAP bio-additive with respect to carbonate-based electrolytes, DFT geometry optimization calculations were performed on the Materials Studio using the Dmol3 application. The calculation parameters are as follows: gga (pW91) density functional keeping spin polarization restricted and B3LYP density functional theory with the 6–311 + + G (d,p) basis set. Figure 1 shows HOMO–LUMO band energy comparison between the electrolyte components ethylene carbonate (EC), diethyl carbonate (DEC), and the bio-additive DMBAP with their DFT energy and geometry optimized structures, respectively. From the results obtained after theoretical evaluation of electrolyte species (EC and DEC) and the DMBAP, it was noted that the HOMO band energy in the case of DMBAP (E_HOMO_ = − 4.40 eV) was higher than that of electrolyte components (E_HOMO_ for EC = − 6.89 and E_HOMO_ for DEC = − 6.52 eV). The higher HOMO band energy of DMBAP suggests that it can readily exhibit the oxidative response on the surface of the cathode prior to the electrolyte species. This would empower the DMBAP to be eligible to undergo in situ oxidative decomposition because of the presence of terminal amine groups during the oxidation half-cycle to form a protection layer on the cathode surface. This phenomenon of sacrificial in situ decomposition of DMBAP would tentatively suppress the following decomposition of electrolyte components on the surface of the cathode. Hence, the theoretical study indicated that the electronic structure of DMBAP can be influencing in the formation of cathode electrolyte interface (CEI) by restricting and optimizing the otherwise uncontrolled decomposition of electrolyte species. Supplementary Table S1 compares the HOMO–LUMO band energies of different electrolyte species (EC and DEC), lithium salts (LiPF_6_ and LiPO_2_F_2_)^37^, various additives (FEC, BIANODA, VC, VEC, PMC, DTD, BOB, and trifluoromethyl-sulfonyl-imide)^32,38^ with the bio-derived DMBAP molecule. To further substantiate the influence of HOMO band energy in pro-longing the cathode cyclability as well as performance while operating at high voltage, the following sections details the electrochemical evaluation of fabricated cathodic half-cells.

### Electrochemical evaluation of DMBAP as an additive

#### Linear sweep voltammetry (LSV) and X-ray photoelectron spectroscopy (XPS) studies

**Figure 2:**
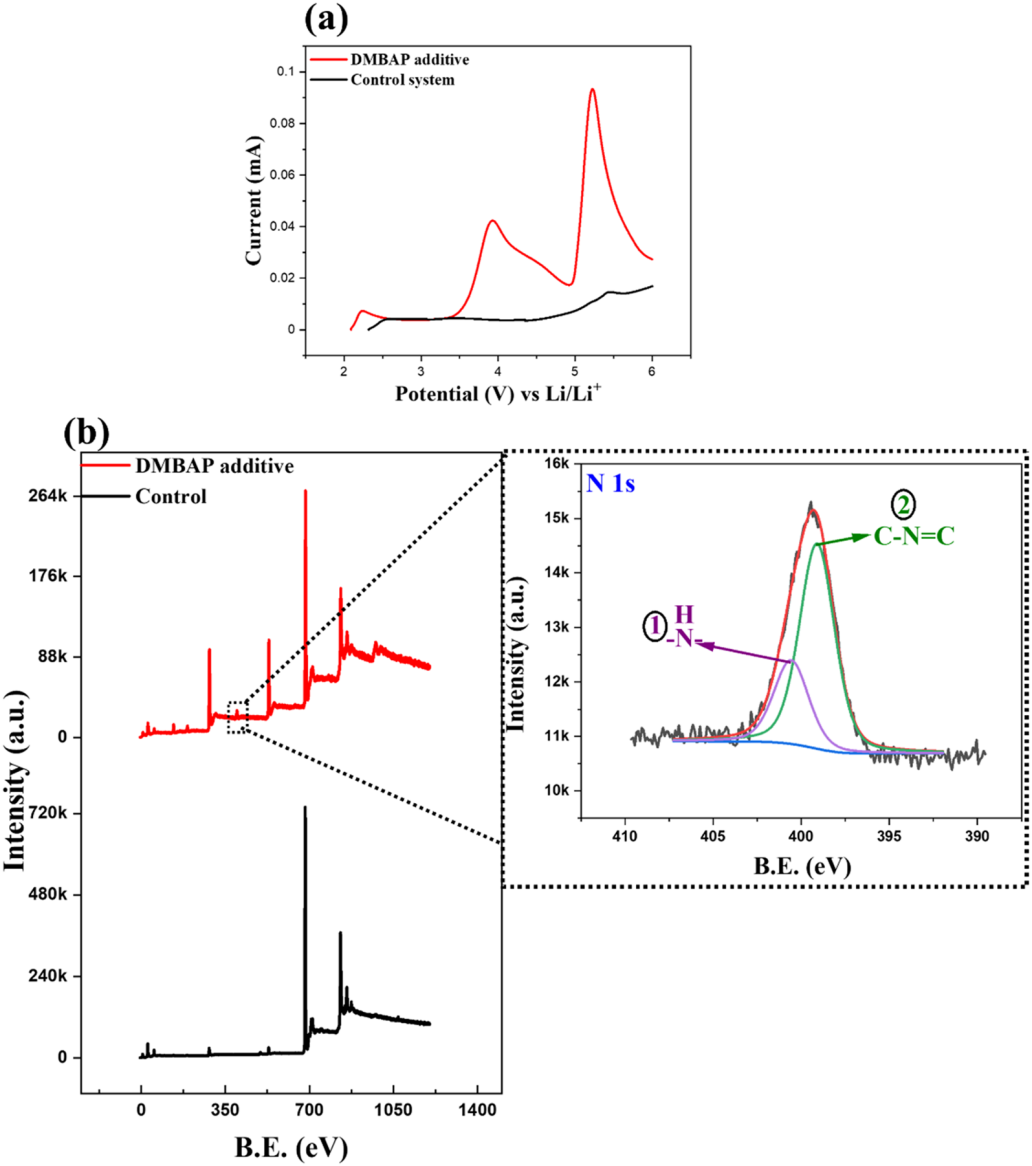
LSV studies and XPS characterization after LSV studies. (**a**) Oxidative linear sweep voltammogram from 0.0 to 6.0 V versus Li/Li^+^ at a scan rate of 1.0 mVs^−1^ and (**b**) XPS spectra recorded after LSV measurements to determine the fate of DMBAP additive after oxidative decomposition in comparison to the control system.

To understand the oxidative and reductive decomposition behaviour of DMBAP, linear sweep voltammetry (LSV) measurements were carried out. Figure 2a shows that the oxidative current in the case of electrolyte containing 2 mg ml^−1^ of DMBAP was much higher in comparison to the control system (reference electrolyte without additive). This oxidative response of the electrolyte system having the DMBAP was in good agreement with the theoretical calculations showing high HOMO band energy for DMBAP bio-additive. In addition, X-ray photoelectron spectroscopy (XPS) technique was used to understand the fate of DMBAP after LSV measurements. Figure 2b shows XPS survey spectra corresponding to DMBAP and control system with a zoomed-in spectrum corresponding to N 1s peak in the DMBAP after oxidative decomposition. Two characteristic peaks in N 1s spectrum of DMBAP were observed with attribution as follows: (i) 398.9 eV corresponding to C−N=C framework^39^, and (ii) 400.4 eV corresponding to terminal -NH_2_ functional group^40^. The peak corresponding to C−N=C framework establishes the fact that DMBAP readily undergoes oxidative electropolymerization to form a passivation layer.

Typically, CEI comprises of inner layer with rigid decomposed inorganic components and soft mesoporous outer layer with decomposed organic components of the electrolyte^41^. Upon electrolyte decomposition at a higher potential, the CEI is usually enriched with organic soft layer with ROCO, ROR, RCO_3_, and P_x_O_y_F_z_ species that promote ionic conductivity^42^. However, at lower potential, the electrolyte decomposition leads to the formation of a CEI that is rich with inorganic species like Li_2_CO_3_, LiF, and Li_x_PF_y_ whose excess formation would obstruct an effective ion transport^41^. As suggested by the theoretical studies of DMBAP and proven by oxidative scan during LSV measurements, the presence of DMBAP in the electrolyte must drive the formation of CEI that is rich with organic species because the sacrificial response of DMBAP would increase the potential window of electrolyte decomposition. Supplementary Fig. S1a–j show the XPS spectra of the corresponding elements C 1s, O 1s, P 2p, Li 1s, and F 1s for DMBAP-based and reference electrolyte (control) systems after LSV measurements. Supplementary Table S2 tabulates their designated deconvoluted elemental peaks with specific percentage area under the curve. The DMBAP-based electrolyte system had larger area under the curve corresponding to organic components attributed to C=O (carbonates, 285.9 eV) and C–O (ethers, 289.3 eV) in C 1s^43^, C–O–C (ethers, 531.4 eV) in O1s^43^, and P=O (phosphates, 136.8 eV) in P 2p, respectively^44^. While the inorganic salt majorly LiF (attributed to Li-F in F 1s–684.6 eV and Li 1s–53.0 eV)^45^ was significantly lesser in the DMBAP-based electrolyte system in comparison to the control system. The excessive deposition of inorganic salts is generally considered toxic to the electrode–electrolyte interphace as its deposition on the electrode surface not only blocks the active site but also it plagues the efficient ionic conduction through the CEI^46–48^. Hence, XPS analysis of the electrolyte species in the presence and absence of DMBAP showed that even small quantity of it can improve the electrochemical stability of carbonate-based electrolytes and influence the formation of CEI.

#### Cyclic voltammetry (CV) and charge–discharge studies

For the electrochemical evaluation, two types of cathodic half-cells were fabricated with LiNi_1/3_Mn_1/3_Co_1/3_O_2_ as the working electrode and Li metal as counter & reference electrode. (1) Cathodic half-cell with 2 mg ml^−1^ of DMBAP solubilized in 1.0 M LiPF_6_ in (50/50) (v/v) EC:DEC, and (2) cathodic half-cell with 1.0 M LiPF_6_ in (50/50) (v/v) EC:DEC electrolyte without any additive (control system).

Supplementary Fig. S2a,b show the cyclic voltammograms of the control system and DMBAP-based cathodic half-cells recorded in the potential window 3.0–4.5 V versus Li/Li^+^. Though the cycling behaviour from second scan onwards was seen similar in both systems, different passivation behaviour was evident only in the first scan of both systems. In the first scan, the DMBAP-based cathodic half-cell showed a positive shift of 0.12 V versus Li/Li^+^ when compared with the control system. This could be attributed to the oxidative response of DMBAP at the interphase of cathode as shown earlier even during LSV studies. Hence this should have significantly contributed to the improvement in the long cycling behaviour of cathodic half-cell with DMBAP additive as discussed later. Also, control and DMBAP-based cathodic half-cells exhibited similar redox peaks corresponding to Ni^2+^/Ni^4+^ at ~ 3.68 V/~ 3.8 V versus Li/Li^+^, respectively^49^. To explore the redox chemistry at even higher potential, cyclic voltammograms of respective cathodic half-cells were recorded in the potential window 3.0–4.8 V versus Li/Li^+^ (Supplementary Fig. S2c,d). Two pairs of typical redox peaks were observed in the control and DMBAP-based cathodic half-cell: (a) attributable to Ni^2+^/Ni^4+^ (~ 3.6 V/3.8 V vs Li/Li^+^), and (b) attributable to Co^2+^/Co^3+^ at a higher potential (~ 4.5 V/4.7 V vs Li/Li^+^), respectively^49^.

**Figure 3:**
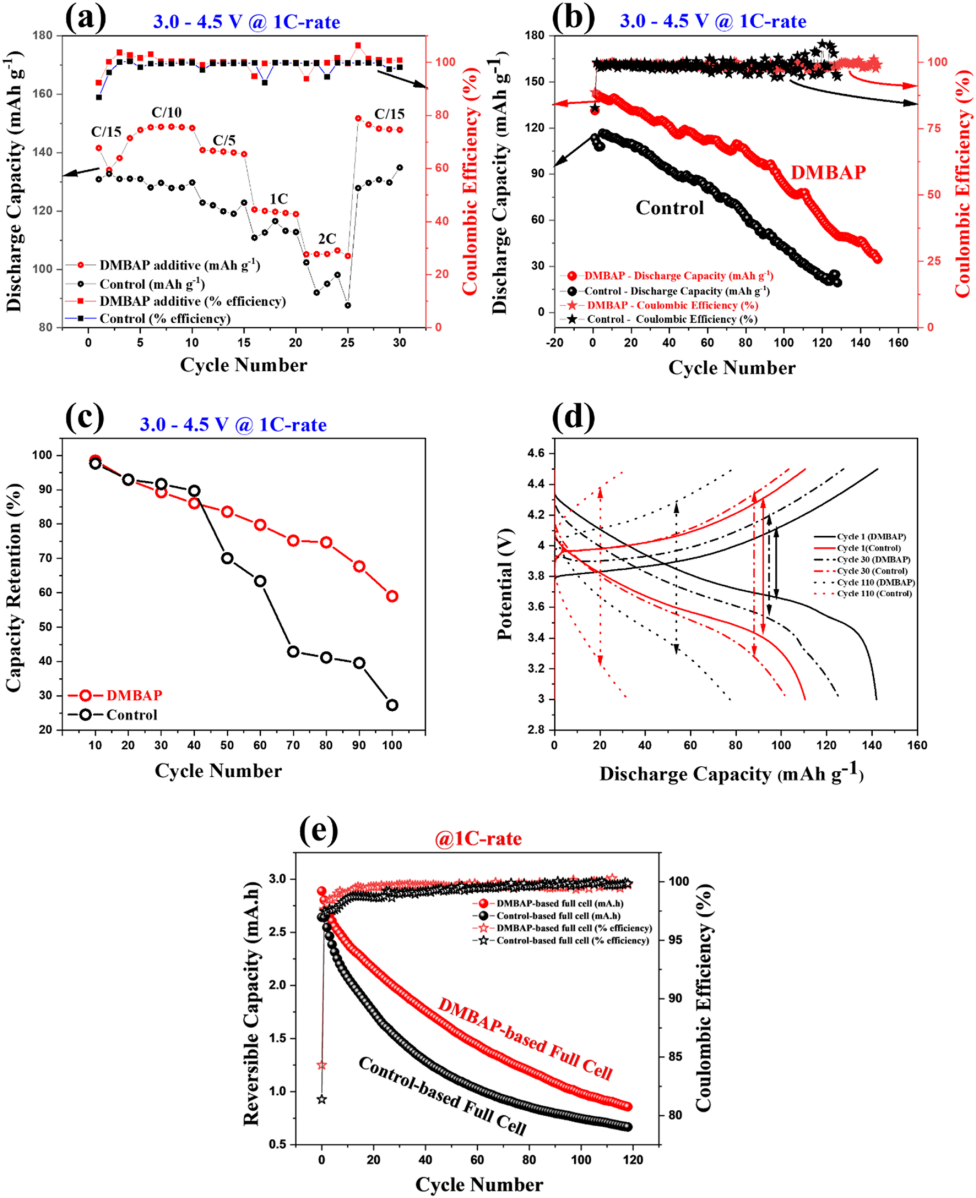
Charge–discharge studies. (**a**) Comparison between charge–discharge performance of control system and DMBAP-based cathodic half-cell at varying current-rates (rate studies), (**b**) long cycling performance of DMBAP-based cathodic half-cell at 1C rate against the control system with no additive, (**c**) comparison of capacity retention between the DMBAP additive-based half-cell and control system, (**d**) comparison of overpotential between the control system and DMBAP-based cathodic half-cells during long cycling at 1C-rate, and (**e**) reversible capacity versus cycle number comparison between DMBAP-based and control electrolyte-based full cells, respectively.

To evaluate the influence of DMBAP in stabilizing LiNi_1/3_Mn_1/3_Co_1/3_O_2_ cathode in terms of rate capability, cyclic stability, coulombic efficiency, and capacity retention, cathodic half-cells with DMBAP and control system (no additive) were subjected to charge–discharge studies. Supplementary Fig. S3a shows the charge–discharge profiles of cathodic half-cells with varying additive content (no additive, 2 mg ml^−1^, 4 mg ml^−1^, and 6 mg ml^−1^) in 1.0 M LiPF_6_ (50/50) (EC:DEC) electrolyte in the potential window 3.0–4.5 V versus Li/Li^+^. The cathodic half-cell with the DMBAP concentration 2 mg ml^−1^ was found to be the best performer towards stabilizing the cathodes. Hence, hereafter, for all the studies, 2 mg ml^−1^ of DMBAP was utilized. Figure 3a compares the performance of cathodic half-cells with DMBAP and control system at varying current rates (C/15, C/10, C/5, 1C, and 2C) and their potential vs capacity curves at various rates are shown in Supplementary Fig. S3b,c, respectively. At current rates C/15 and C/10, cumulatively, the DMBAP-based cathodic half-cell showed an average reversible capacity of 145 mAh g^−1^ with an initial coulombic efficiency (ICE) of ~ 92.3% in comparison to the control system that showed 128 mAh g^−1^ with an ICE ~ 86.8%. In the case of DMBAP-based cathodic half-cell, the higher ICE signifies lower irreversible capacity due to the restricted electrolytic oxidative decomposition. This was consistent with theoretical and LSV studies that indicated that DMBAP upon oxidative decomposition would curb the extent of electrolyte decomposition. As a result, the DMBAP-based cathodic half-cell outperformed the control system exhibiting an excellent rate-capability with ~ 25.3% higher average reversible capacity and ~ 1.6% higher coulombic efficiency (CE) throughout. Therefore, to compare the cyclic stability of LiNi_1/3_Mn_1/3_Co_1/3_O_2_ cathode under the influence of DMBAP and without any additive (control), long cycle charge–discharge studies were carried out at 1C current rate. As shown in Fig. 3b, the cathodic half-cell with DMBAP showed improved cyclic stability (~ 150 cycles) with a reversible capacity of 83.34 mAh g^−1^ after 100 cycles in comparison to the control system (42.6 mAh g^−1^ after 100 cycles). Hence, the DMBAP additive improved the specific capacity of LiNi_1/3_Mn_1/3_Co_1/3_O_2_ cathode by ~ 49% in comparison to the control system. As shown in Fig. 3c, the cathodic half-cell with DMBAP showed 59% capacity retention after 100 cycles of charge–discharge against the control system showing only 27.3%. In addition, throughout 150 cycles of charge–discharge, the DMBAP-based cathodic half-cell showed 55.6% higher reversible capacity in comparison to the control system. Supplementary Fig. S3d shows the comparison of the coulombic efficiency of cathodic-half-cells with DMBAP and control system for long cycling at 1C. At 1C-rate, the ICE of the DMBAP-based cathodic half-cell was observed to be 88.7% against the control system with 82.8%. Also, the control system exhibited a variable CE with poor stability in comparison to the DMBAP-based cathodic half-cell. The improved capacity retention and cyclic stability of the cathodic half-cell with DMBAP bio-based additive against the control system were also supported by the voltage-capacity curves (Supplementary Fig. S3e,f). Evidently, the voltage decay in the case of the control system was severe and rapid against the DMBAP-based cathodic half-cell that showed better stability in discharge plateaus. Also, significant drop in the overpotential (Fig. 3d) throughout long cycle charge–discharge process was observed in the case of DMBAP-based cathodic half-cell in comparison to the control system because of passivation layer formation on the LiNi_1/3_Mn_1/3_Co_1/3_O_2_ cathode by the DMBAP bio-based additive. Hence, a small quantity (2 mg ml^−1^) of bio-base additive DMBAP enhanced the performance of LiNi_1/3_Mn_1/3_Co_1/3_O_2_ cathodes in terms of operating potential limit (4.5 V vs Li/Li^+^), rate capability, capacity retention, irreversible capacity loss (coulombic efficiency), and reversible specific capacity. In addition, to evaluate the overall full-cell performance and the influence of DMBAP as additive to stabilize the cathode, full cells fabricated with DMBAP-based electrolyte (2 mg ml^−1^) and control (no additive) were evaluated. Galvanostatic charge–discharge profiles of respective full cells are shown in Figs. 3e and S4, respectively. In comparison to the control full cell (no additive) that showed poor reversible capacity retention of ~ 25.3% after 118 cycles of charge–discharge, the full cell with DMBAP additive showed higher capacity retention of ~ 29.7%. Also, the full cell with DMBAP additive reached > 99% CE within first 7 cycles in comparison to the control full cell that took 25 cycles indicating higher irreversible capacity loss owing to the undesirable excessive electrolyte decomposition on the electrode. In the first positive scan (charging of full cell or delithiation of cathode) from 2.0 to 4.5 V versus Li/Li^+^, plateau corresponding to DMBAP oxidation on the cathode was evident in contrast to the control full cell with no additive. This further explains the cyclic stability and higher coulombic efficiency of the full cell with DMBAP additive as DMBAP’s sacrificial oxidation inspired protection layer formation on the cathode that armours it for better cyclic performance and stability.

#### (Dynamic) electrochemical impedance spectroscopy

**Figure 4:**
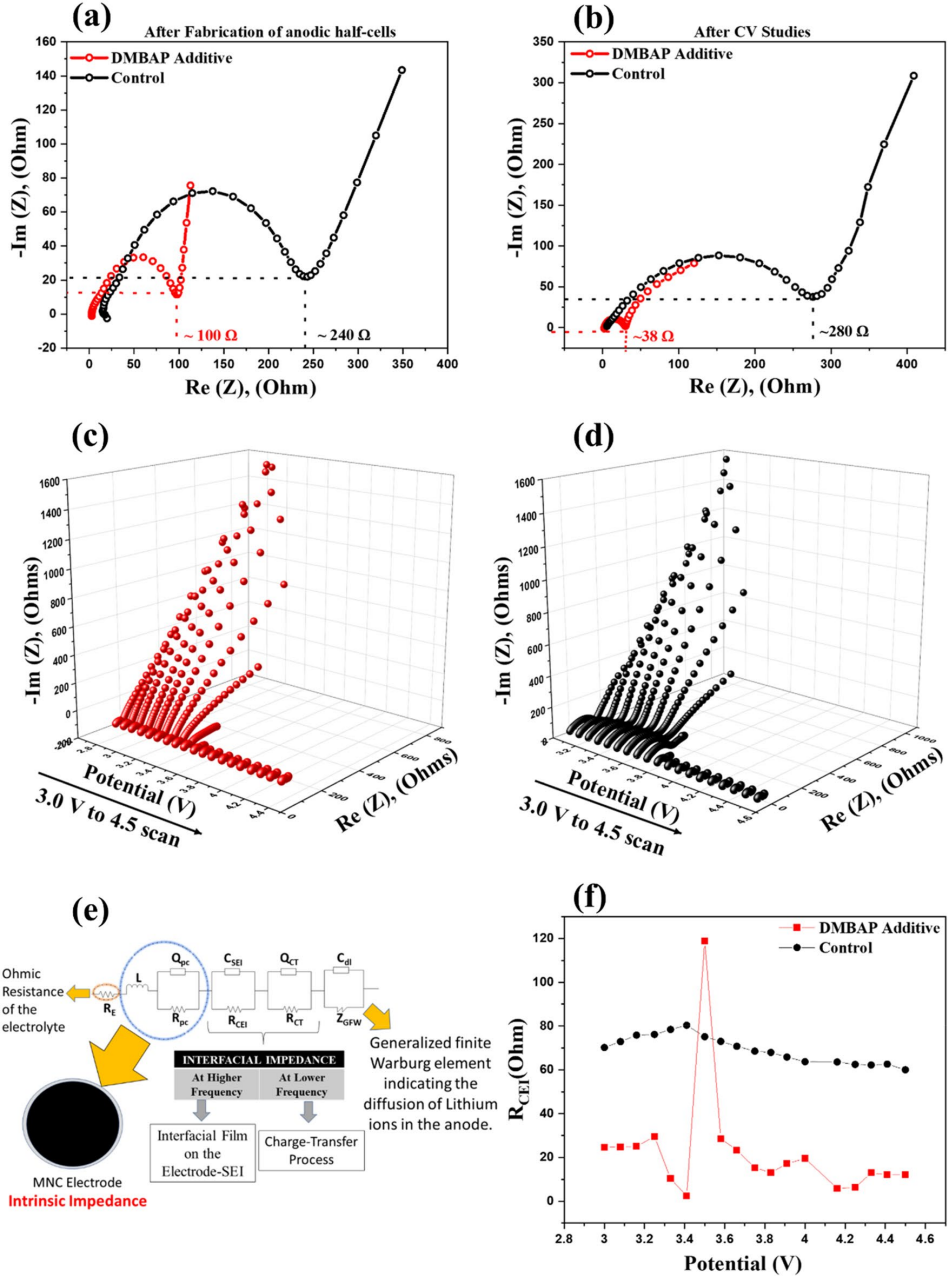
Impedance Spectroscopy Studies. Nyquist impedance profile comparison for DMBAP-based cathodic half-cell and control system (**a**) after fabrication and (**b**) after CV studies, respectively. DEIS 3-D Nyquist profiles after delithiation half-cycle of (**c**) DMBAP-based cathodic half-cell and (**d**) control system-based cathodic half-cell. (**e**) The best fit Equivalent Electrical Circuit Model (EECM) for computational simulation of 3D-Nyquist Impedance profiles for both systems, and (**f**) CEI impedance (R_CEI_) versus potential (V) comparison profiles during lithiation half-cycle in case of DMBAP-based cathodic half-cell and control system, respectively.

To understand the properties of the CEI formed during the cycling of cathodic half-cells, electrochemical impedance spectroscopy (EIS) studies were carried out. The EIS measurements were recorded without direct current (DC) at the open circuit potential (OCP) of respective cathodic half-cells. Figure 4a,b show the Nyquist impedance profiles of DMBAP and control system-based cathodic half-cells recorded after their fabrication and post-cyclic voltammetry (CV) studies, respectively. The internal impedance after fabrication in the case of the control system was observed to be ~ 240 Ω. However, the DMBAP-based cathodic half-cell showed lower internal impedance (~ 100 Ω). After cyclic voltammetry (CV) studies, the impedance of the DMBAP-based cathodic half-cell decreased to 38 Ω constituting of impedance contribution by CEI and charge-transfer (CT) (R_CEI_ + R_CT_) in comparison to the control system that had a rise in its impedance to 280 Ω (R_CEI_ + R_CT_). Unlike the DMBAP-based cathodic-half cell, the increase in the internal impedance of the control system after CV studies was attributed to the process of uncontrolled oxidative decomposition of electrolyte species leading to a CEI with an increase in the internal impedance. However, EIS studies when carried out at OCP did not provide deeper insights into the types of interphases and the corresponding impedance values during a real-time charge–discharge of the cathodic half-cell. Therefore, to obtain a comprehensive understanding about the types of interfaces and their contribution to the total cell impedance, an advanced impedance spectroscopy technique—dynamic electrochemical impedance spectroscopy (DEIS) was employed. In the DEIS technique, the impedance profiles were recorded at different potential steps during charge–discharge processes. Here, the frequency response of cathodic half-cells was recorded corresponding to an AC signal in the operating potential window (3.0–4.5 V vs Li/Li^+^). Figure 4c,d show the 3D-Nyqiust impedance profiles recorded during the delithiation of DMBAP and control system-based cathodic half-cells after 100 cycles. Similarly, Supplementary Fig. S5a,b show the 3D-Nyqiust impedance profiles during recorded during the lithiation of DMBAP and control system-based cathodic half-cells after 100 cycles. To understand the types of interphases that are formed during the electrochemical evaluation of cathodic half-cells, the recorded Nyquist profiles from the DEIS studies were simulated computationally with various probable Equivalent Electrical Circuit Models (EECMs). As shown in Fig. 4e, the best fit EECM was (RL(QR)(QR)(QR)(CW)). A representative Nyquist profile of LiNi_1/3_Mn_1/3_Co_1/3_O_2_ cathode with DMBAP fit with the EECM at 4.0 V versus Li/Li^+^ is shown in Supplementary Fig. S5c. Each element in the electrical circuit model was attributed as follows: Ohmic electrolytic resistance (R_e_), intrinsic resistance of the LiNi_1/3_Mn_1/3_Co_1/3_O_2_ cathode comprising of the particle to particle and particle to current collector (R_PC_), cathode-electrolyte interface (R_CEI_), charge transfer resistance (R_CT_), and Warburg infinite diffusion element (Z_GFW_). Supplementary Tables S3 and S4 list the values of respective circuit fitting parameters (resistance corresponding to each of the interphases involved) as per the EECM for DMBAP and control system-based cathodic half-cell. Figure 4f compares the CEI resistance (R_CEI_) corresponding to DMBAP-based and control system against the potential steps during the lithiation half-cycle. The R_CEI_ corresponding to the DMBAP-based cathodic half-cell was substantially lower than the control system at all potential points. Therefore, the DEIS study elaborated the influence of passivation layer formed by the DMBAP to supress the extent of electrolyte decomposition and tailor a CEI with lower interfacial resistance.

### Post-mortem characterization

#### Scanning electron microscope (SEM) measurements (Morphology studies)

**Figure 5:**
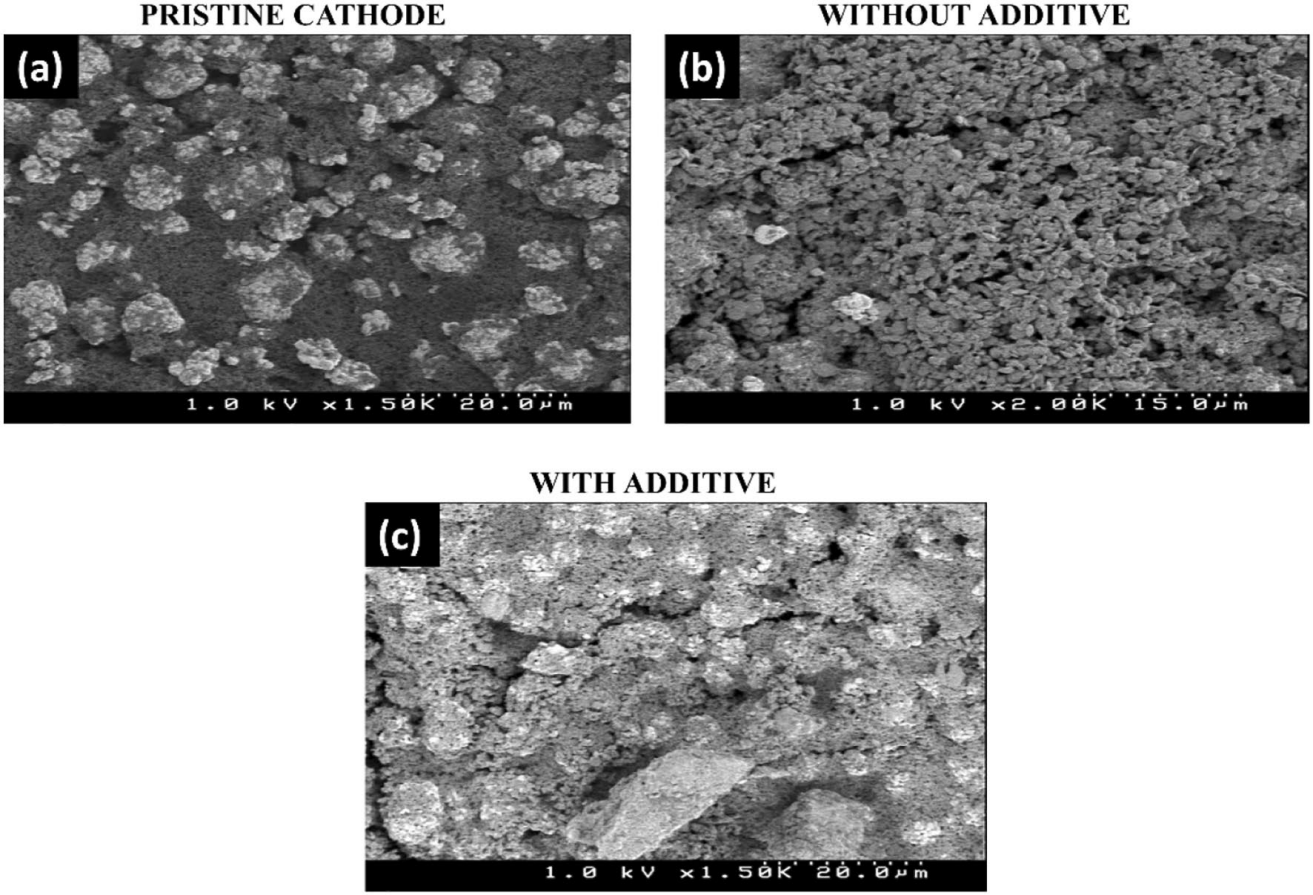
Study of surface morphology of LiNi_1/3_Mn_1/3_Co_1/3_O_2_ cathodes upon storage in electrolyte. SEM micrographs of (**a**) pristine cathode, (**b**) cathode stored in electrolyte with no additive, and (**c**) cathode stored in electrolyte with DMBAP additive, respectively.

Doeff et al. showed that the surface of transition metal-based LIB cathodes undergoes corrosion upon their prolonged exposure to electrolytes^19^. It is because of the detrimental reaction that take place between transition metals and the lithium salt in the electrolyte^19^. This results in gradual increase in the impedance while operating at a high voltage^19^. Therefore, the real-time operation of LiNi_1/3_Mn_1/3_Co_1/3_O_2_ cathodes in carbonate-based electrolyte over a long-term has been troublesome. Hence, to investigate the effect of DMBAP in moderating the interactions between LiNi_1/3_Mn_1/3_Co_1/3_O_2_ cathodes and electrolyte, two systems were prepared and studied as follows: storing the cathode in commercial 1.0 M LiPF_6_ (50/50) (EC:DEC) electrolyte (a) with DMBAP additive (2 mg ml^−1^) and (b) without additive for seven days in argon atmosphere at a dark place at room temperature. SEM micrographs of the pristine electrode, electrode stored in the electrolyte without additive, and electrode stored in the electrolyte with DMBAP additive are represented in Fig. 5. The pristine cathode showed micrometre size spherical particles composed of bead like morphology present in a uniform matrix of conductive additive. Upon storage in the electrolyte without additive, the primary bead like morphology was disintegrated into unclear substructure and surface matrices showed a turbid deposition of the surface reaction layer with corrosion. However, the cathode stored in the electrolyte with DMBAP additive retained its primary bead like morphology to a large extent. Therefore, the presence of DMBAP additive even in low concentration redeemed the interaction of cathode surface with the electrolyte.

#### X-ray photoelectron spectroscopy (XPS) studies

As XPS studies provide comprehensive insights into the quantitative details of CEI composition, XPS spectra of pristine LiNi_1/3_Mn_1/3_Co_1/3_O_2_ cathode, cathode after 100 cycles of charge–discharge with DMBAP additive, and without additive (control) were recorded. Regarding this, respective cathodic half-cells after charge–discharge studies were disassembled in an argon filled glovebox. After disassembling the cathodic half-cells, retrieved cathodes were washed multiple times with fresh electrolyte solvent, and dried under vacuum condition for over 15 h. The XPS measurement was carried while maintaining completely air-free condition with ultra-high vacuum of ~ 5.0 × 10^−7^ Pa. The measurement parameters were as follows: dwell time—100 ms, number of scans—5, step size—1 eV for survey, and 0.1 eV for elemental scans. The survey spectra are shown in Supplementary Fig. S6. During cycling, in the first oxidation half-cycle at the LiNi_1/3_Mn_1/3_Co_1/3_O_2_ cathode, the DMBAP underwent oxidative decomposition to form a passive layer on the cathode followed by the electrolyte decomposition. Hence, the detection of N 1s peak in the survey spectrum of the DMBAP-based cathode indicated that the CEI formed was thin as the detection depth of X-rays is ~ 5–8 nm. Further, individual elements from respective cathodes (pristine, DMBAP-based and control) were resolved and deconvoluted. Their C 1s and O 1s spectra are shown in Supplementary Fig. S7. In the C 1s spectra, the pristine electrode showed characteristic peaks attributed to C–C (284.6 eV), C–O (285.6 eV), and CF_2_ (290.4 eV) corresponding to active materials/conductive additive, adsorbed oxygen moieties on the surface of cathode, and fluoride-based binder^50,51^. Also, the presence of a weak C=O (287.3 eV)^51^ was indicative of presence of surface films formed by the reaction with CO_2_ with moisture to form lithium carbonate (Li_2_CO_3_). However, after the electrochemical evaluation, carbonate (C=O, 287.2 and 288.0 eV)^51^ was observed as an electrolyte decomposition product on the cathode surface in the case of control and DMBAP-based systems^51^. In the O 1s spectra, the pristine electrode showed a prominent lattice oxygen peak (531.6 eV), transition metal oxides peak (529.9 eV), and the decomposed carbonate species (534.9 eV). However, after cycling, in the case of control system (without additive), the intensity of characteristic lattice oxygen peak was lower in comparison to DMBAP-based system^50,51^. This could be due to the restricted decomposition of the electrolyte as the passivation layer formed by the DMBAP masked metal centres and surface of the cathode. Supplementary Fig. S8 shows the F 1s, P 2p, and N 1s spectra of respective cathodes. Peaks corresponding to the formation of Li-F/P-F decomposed products (685.9 eV) on the electrode are comparable in both systems^50^. However, the intensity of C–F peak (689.2 eV) attributable to the binder (PVDF) in LiNi_1/3_Mn_1/3_Co_1/3_O_2_ cathode matrix was less intense in the case of control system than the DMBAP-based cathode, indicating that the CEI formed on the control system was thicker. In the P 2p spectra, peaks corresponding to P–O/P=O decomposed products were observed in both systems^50^. Most importantly, the presence of two characteristic peaks (C−N=C framework and terminal –NH_2_) in N 1s spectrum of DMBAP-based cathode corresponds to decomposed DMBAP. Hence, the detection of constitutional peaks in the DMBAP-based cathode like C–F of the PVDF binder in the electrode laminate in C 1s and F 1s spectra, lattice oxygen peaks, and nitrogen peaks corresponding to the DMBAP bio-based additive indicated that the CEI thickness was optimal for the XPS analysis of the core electrode laminate.

## Conclusion

A microbially derived 2,5-dimethyl-3,6-bis(4-aminobenzyl)pyrazine (DMBAP) compound was investigated for its ability to act as electrolyte additive to stabilize high-voltage LiNi_1/3_Mn_1/3_Co_1/3_O_2_ cathodes by exploiting its structural and electrochemical behaviour in oxidative environment in a LIB. By the theoretical evaluation of DMBAP, it was found that its HOMO band energy was significantly higher than that of commercial electrolyte components. This inspired its detailed electrochemical evaluation as an additive in LiNi_1/3_Mn_1/3_Co_1/3_O_2_ cathode-based half-cells with techniques like linear sweep voltammetry, cyclic voltammetry, and impedance (including sophisticated DEIS). Further, post-mortem evaluation of the LiNi_1/3_Mn_1/3_Co_1/3_O_2_ cathode after the electrochemical evaluation was studied by FESEM, and XPS techniques. It was concluded that the DMBAP could undergo sacrificial oxidative decomposition to form an organic passivation layer on the cathode surface and suppressed the extent of oxidative decomposition of electrolyte species. This phenomenon proved to be virtuous as it increased the operating potential window while working with LiNi_1/3_Mn_1/3_Co_1/3_O_2_ cathode to 4.5 V versus Li/Li^+^ and stabilized it in terms of cyclic stability, rate capability, reversible capacity, and coulombic efficiency. The highlighting aspect of this study was microbial origin of DMBAP (from the gene cluster of *Pseudomonas fluorescens* SBW25). This provided a green footprint in LIB technology development and an inspiration to explore more sustainable, eco-friendly, and cost-effective alternatives.

## Experimental section

### Materials, cell assembly and electrochemical measurements

For the electrochemical characterization, commercial LiMn_x_Ni_y_Co_z_O_2_ (x = y = z = 1/3) electrodes were purchased from Piotrek, Japan. The reported reversible capacities in this study are based on the total electrode weight (not the active material weight). 1.0 M LiPF_6_ in (50/50) (v/v) EC:DEC was used as the electrolyte (purchased from Sigma Aldrich). DMBAP was fermented by using recombinant *Escherichia coli* cells and purified by the procedure reported by Masuo et al.^33^ Briefly, the *E.coli* NDG strain expressing papABCDEF was cultured in 1-kL jar fermenter containing 600 L of fermentation medium for 48 h at 30 °C at 355 rpm. DMBAP in acidified culture supernatant (pH 3.5, 600 L) was purified by cation exchange resin (DIAIONTM PK212LH, Mitsubishi Chemical Corporation). The resin was washed with water and methanol, and then DMBAP was eluted with ethanol containing 5% NH_3_. The eluent was concentrated, and then underwent diethyl ether extraction and recrystallization to obtain purified DMBAP (purity > 96%). Vacuum dried DMBAP was added to the commercial LiPF_6_ electrolyte to evaluate its performance as an additive. To understand the oxidative decomposition characteristics of electrolyte species with and without DMBAP additive, linear sweep voltammetry (LSV) measurements of the control system (without additive) and DMBAP-based system were recorded between 0 and 6 V versus Li/Li^+^. And, to evaluate the reductive decomposition response, the LSV measurements were recorded between open circuit potentials (OCPs) and 0 V versus Li/Li^+^ for the corresponding systems. The LSV measurements were recorded at a scan rate of 1 mVs^−1^. For, LSV measurements, test cells were fabricated with the following cell assembly: polypropylene separator (25 mm, Celgard) sandwiched between stainless steel disc as working electrode and Li metal as counter and reference electrode. For the electrochemical evaluation of the Li-NMC cathode with and without DMBAP additive, 2025-type coin cells were fabricated with the following configuration: Li-NMC as cathode, polypropylene separator (25 mm, Celgard 2500), additive containing electrolyte/only electrolyte (control system), and lithium metal (Honjo metals) as counter and reference electrode, respectively. The cells were assembled inside an argon-filled glovebox to avoid the moisture contamination (UNICO UN-650F, H_2_O and O_2_ content < 0.1 ppm). The charge–discharge studies were carried at 25 °C on Electrofield-EFT-001. A VSP potentiostat (BioLogic) electrochemical analyzer/workstation was used for the electrochemical characterization of the fabricated half-cells by cyclic voltammetry (CV) measurements between 3.0–4.5 V and 3.0–4.8 V versus Li/Li^+^ at 25 °C at a scan rate of 0.1 mVs^−1^. Electrochemical impedance spectroscopy (EIS) and dynamic electrochemical impedance spectroscopy (DEIS) studies were conducted on a VSP potentiostat (Biologic) within a frequency range of 10 mHz–1 MHz with a sinus amplitude of 10 mV.

### Characterizations

The Fourier transform infrared spectroscopy (FTIR) measurements were recorded using a PerkinElmer 100 FT-IR spectrometer. The spectra were averaged over 100 scans having a resolution of 2 cm^−1^ in the attenuated total reflectance mode. The X-ray photoelectron spectroscopy (XPS) measurements were recorded on a delay-linked detector (DLD) (Kratos Axis-Ultra; Kratos Analytical Ltd.) with an Al Kα radiation source (1486.6 eV). A Hitachi S-4500 field emission scanning electron microscopy (FESEM) instrument was used to obtain the scanning electron microscopy (SEM) images of respective cathodes.

## Supplementary Information

Supplementary Information.

## Data Availability

The data that support the results of this study are available from the corresponding author upon request.
