# Supplementary material for: Microbial pyrazine diamine is a novel electrolyte additive that shields high-voltage LiNi1/3Co1/3Mn1/3O2 cathodes
Source: Sci Rep. 2022 Nov 25;12:19888. doi: 10.1038/s41598-022-22018-1 (PMC9700740; doi:10.1038/s41598-022-22018-1)
Supplement: Supplementary file 1 — Supplementary Information. [file 41598_2022_22018_MOESM1_ESM.docx]

Microbial Pyrazine Diamine is a Novel Electrolyte Additive that Shields High-Voltage LiNi_1/3_Co_1/3_Mn_1/3_O_2_ Cathodes

Agman Gupta^1^, Rajashekar Badam^1^, Noriyuki Takamori^1^, Hajime Minakawa^2^, Shunsuke Masuo^2^, Naoki Takaya^2^, and Noriyoshi Matsumi^1^*

1. Graduate School of Advanced Science and Technology, Japan Advanced Institute of Science and Technology (JAIST), 1-1 Asahidai, Nomi, Ishikawa, 923-1292, Japan

2. Faculty of Life and Environmental Science, Microbilogy Research Center for Sustainability, University of Tsukuba, 1-1-1 Tennodai, Tsukuba, Ibaraki 305-8577, Japan

Email: [matsumi@jaist.ac.jp](mailto:matsumi@jaist.ac.jp)

**Supplementary Table S1:** Comparison of HOMO & LUMO energy levels of the DMBAP additive with common carbonate-based electrolyte components and LiPF_6_ salt.

| Material | HOMO (eV) | LUMO (eV) |
| --- | --- | --- |
| EC | -6.89 | -0.28 |
| DEC | -6.52 | -0.06 |
| LiPF_6_ | -6.13 | -1.52 |
| LiPO_2_F­_2_ | -8.57 | -1.10 |
| FEC | -11.02 | 0.07 |
| BIANODA | -4.71 | -2.06 |
| VC | -8.74 | 0.13 |
| VEC | -9.42 | 0.04 |
| PMC | -9.58 | -0.07 |
| DTD | -10.77 | 0.04 |
| BOB | -9.71 | -0.74 |
| 1,1′-(5,14-dioxo-4,6,13,15-tetraazaoctadecane-1,18diyl) bis(3-(sec-butyl)-1H-imidazol-3-ium) bis((trifluoromethyl)-sulfonyl) imide | -10.10 | -0.27 |
| DMBAP Additive  (This work) | **-4.43**  **(High HOMO)** | **-2.40** |


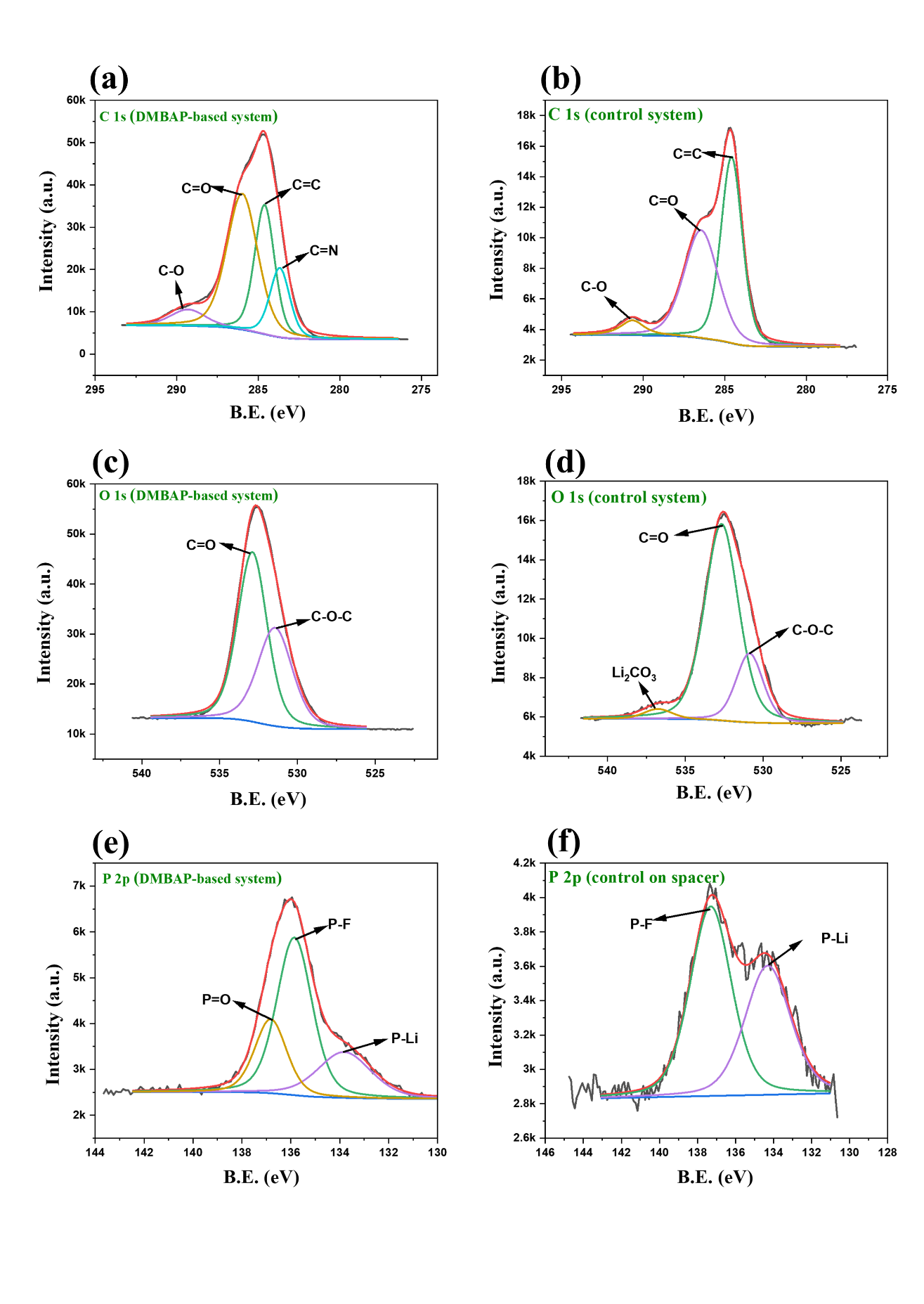


**
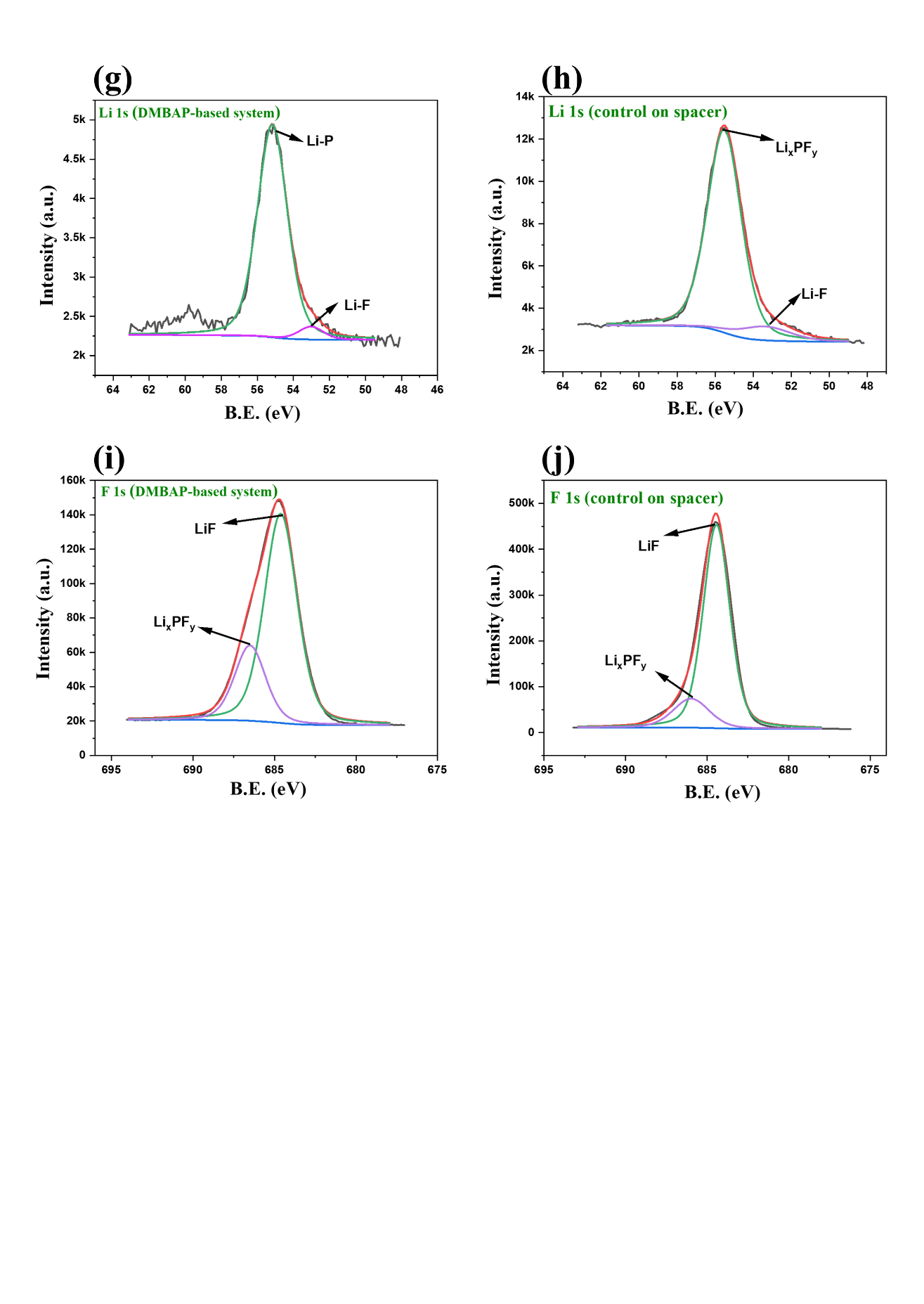
**

**Supplementary Figure S1:** The XPS spectra recorded after the LSV measurements (a), (c), (e), (g), and (i) for the DMBAP-based system corresponding to C 1s O 1s, P 2p, Li 1s, and F 1s, and (b), (d), (f), (h) and (j) for the control system (no additive) respectively.

**Supplementary Table S2:** Tabulated binding energy values of relevant elemental peaks for the electrolyte system with DMBAP additive and control (no additive) after LSV measurements explaining the corresponding % area under the curve for (a) C 1s, (b) O 1s, (c) P 2p, (d) Li 1s, and F 1s, respectively.

(a) C 1s (DMBAP-based and control systems)

| **Component** | **DMBAP-based system**  **(eV)** | **% area under the curve** | **Control (no additive)**  **(eV)** | **% area under the curve** |
| --- | --- | --- | --- | --- |
| C=N | 283.6 | 15.8 | - | - |
| C=C | 284.6 | 29.5 | 284.5 | 49.1 |
| C=O | 285.9 | 48.0 | 286.4 | 45.5 |
| C-O | 289.3 | 6.5 | 290.6 | 5.2 |

(b) O 1s (DMBAP-based and control systems)

| **Component** | **DMBAP-based system**  **(eV)** | **% area under the curve** | **Control (no additive)**  **(eV)** | **% area under the curve** |
| --- | --- | --- | --- | --- |
| C-O-C | 531.4 | 40.4 | 530.8 | 21.3 |
| C=O | 532.9 | 59.5 | 532.5 | 75.5 |
| Lithium carbonates (Li_2_CO_3_) | - | - | 536.7 | 3.1 |

(c) P 2p (DMBAP-based and control systems)

| **Component** | **DMBAP-based system**  **(eV)** | **% area under the curve** | **Control (no additive)**  **(eV)** | **% area under the curve** |
| --- | --- | --- | --- | --- |
| Li-P | 133.8 | 23.6 | 134.3 | 57.0 |
| P-F | 135.8 | 53.6 | 137.3 | 42.9 |
| P=O | 136.8 | 22.6 | - | - |

(d) Li 1s (DMBAP-based and control systems)

| **Component** | **DMBAP-based system**  **(eV)** | **% area under the curve** | **Control (no additive)**  **(eV)** | **% area under the curve** |
| --- | --- | --- | --- | --- |
| LiF | 53.0 | 4.7 | 53.3 | 9.3 |
| Li-P | 55.1 | 95.2 | 55.5 | 90.6 |

(e) F 1s (DMBAP-based and control systems)

| **Component** | **DMBAP-based system**  **(eV)** | **% area under the curve** | **Control (no additive)**  **(eV)** | **% area under the curve** |
| --- | --- | --- | --- | --- |
| LiF | 684.6 | 74.6 | 684.4 | 83.7 |
| Li_x_PF_y_ | 686.5 | 25.3 | 685.9 | 16.2 |


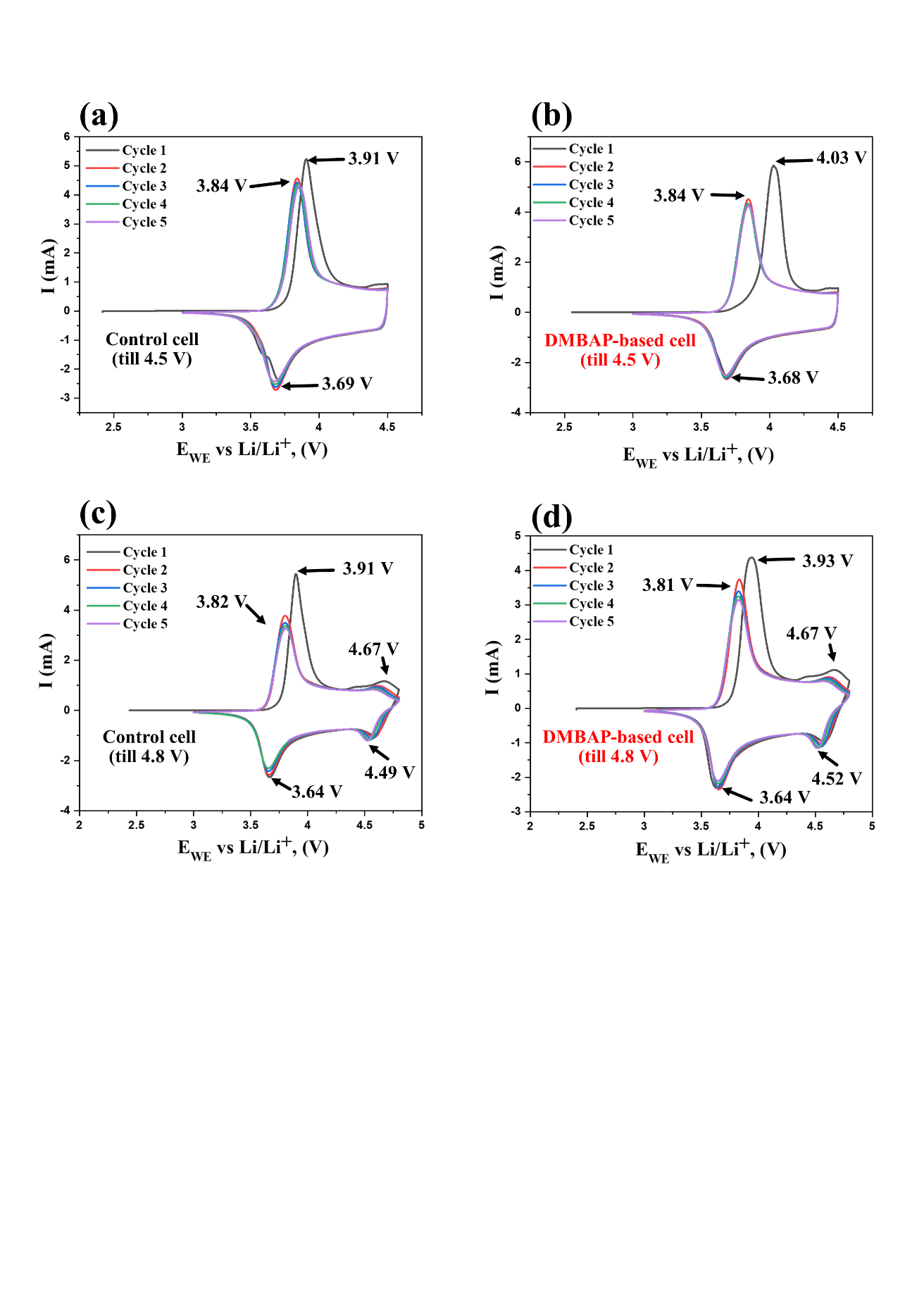


Supplementary Figure S2: Cyclic voltammograms of cathodic half-cells fabricated with DMBAP additive (2 mg ml^-1^ in the electrolyte) and control system (without additive): (a) Control system (3.0 V – 4.5 V vs Li/Li^+^), (b) DMBAP-based system (3.0 – 4.5 V vs Li/Li^+^), (c) Control system (3.0 V – 4.8 V vs Li/Li^+^), and (d) DMBAP-based system (3.0 – 4.8 V vs Li/Li^+^), respectively.


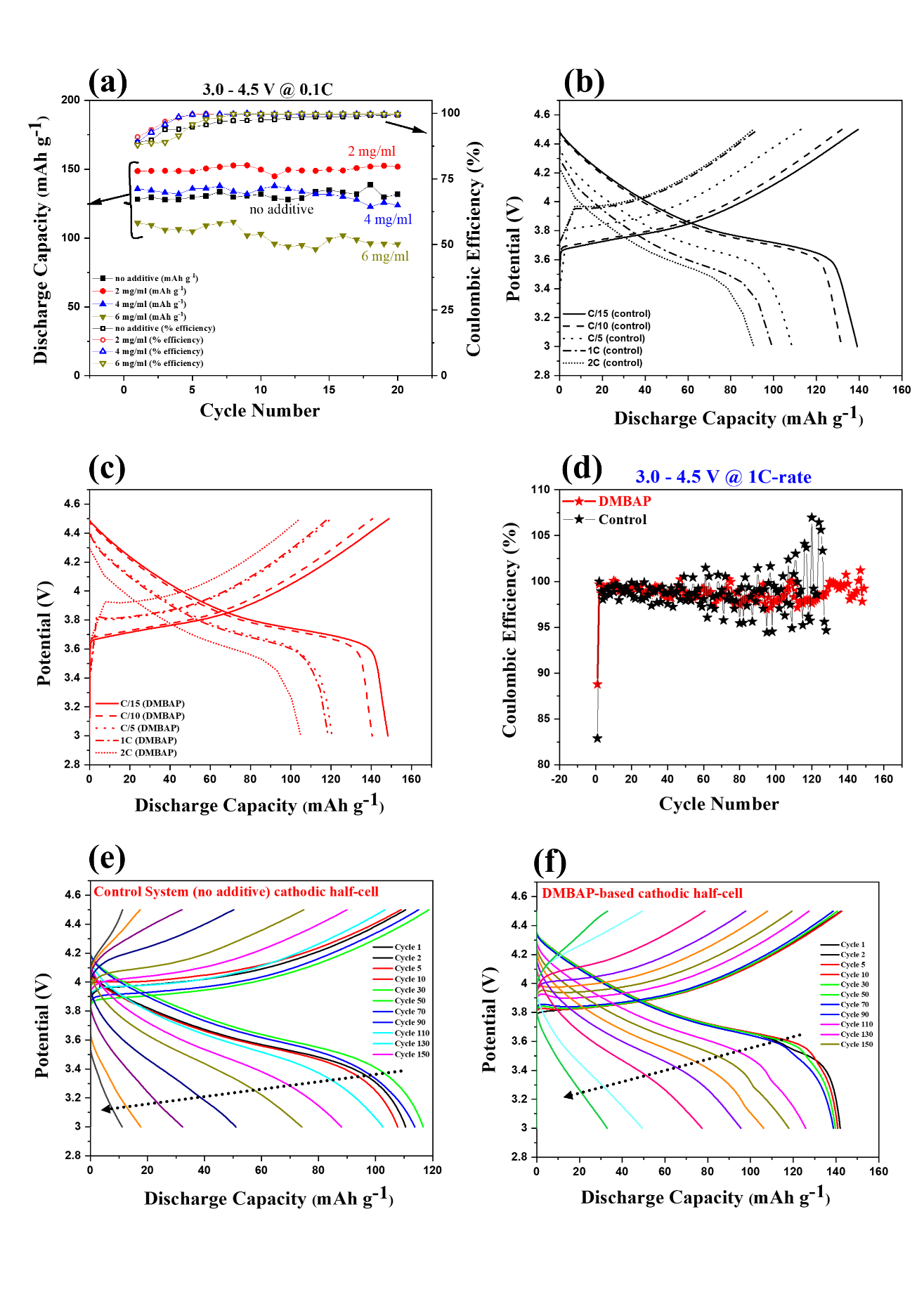


**Supplementary Figure S3:** (a) Charge-discharge profiles of cathodic-half cells fabricated with varying DMBAP amount in the electrolyte, (b) charge-discharge (potential vs capacity) curves of the control system-based cathodic half -cell after the rate studies, (c) charge-discharge (potential vs capacity) curves of the DMBAP-based cathodic half-cell after the rate studies, (d) coulombic efficiency comparison between the control and DMBAP-based cathodic half-cells for long cycling performance at 1C-rate, (e) charge-discharge (potential vs capacity) curves of the control system-based cathodic half -cell after long cycling at 1C-rate, and (f) charge-discharge (potential vs capacity) curves of the DMBAP-based cathodic half-cell after long cycling at 1C-rate.


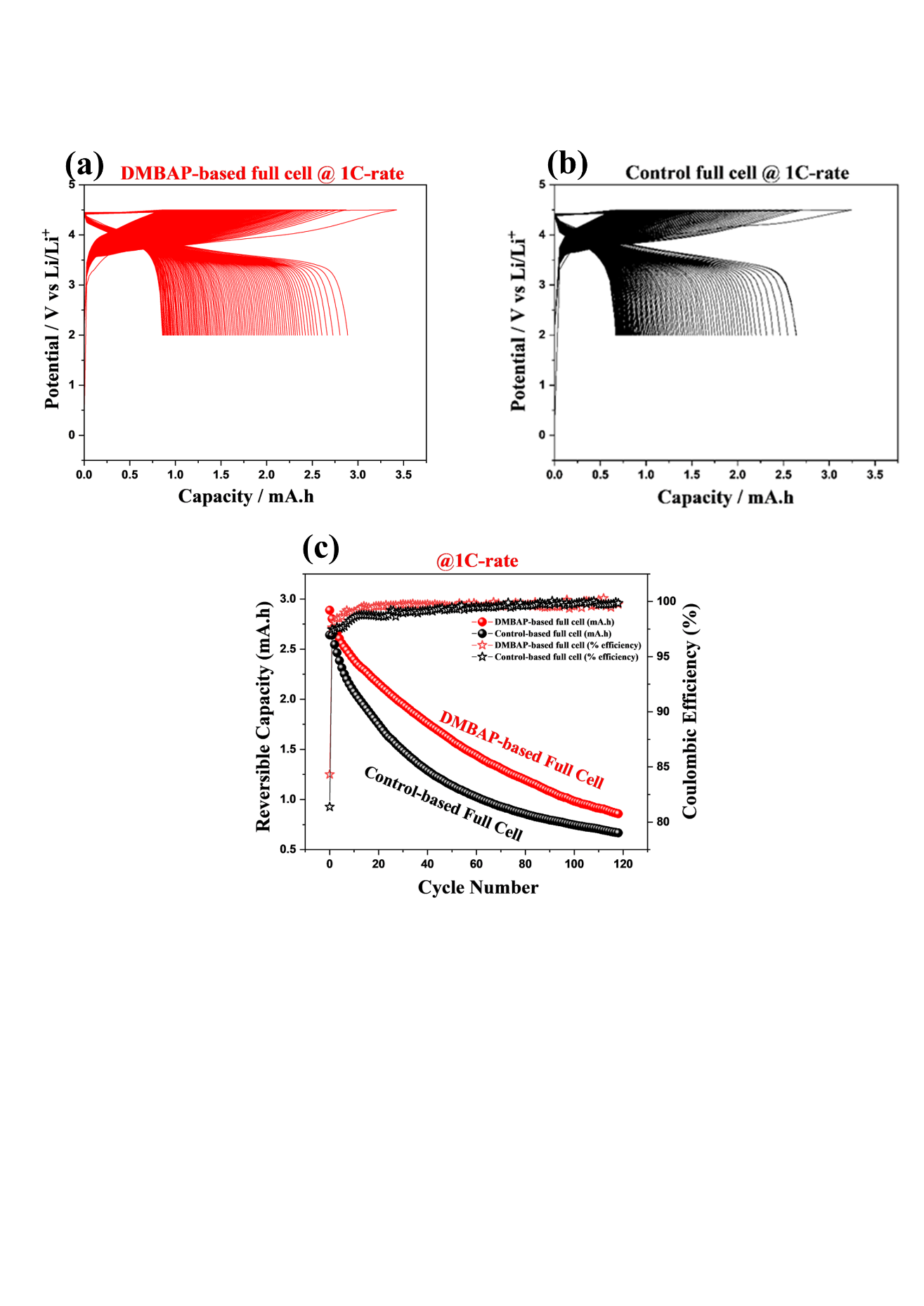


**Supplementary Figure S4:** Galvanostatic charge-discharge profiles of full cells fabricated with (a) DMBAP-based electrolyte (2 mg ml^-1^): potential vs reversible capacity plots and (b) control electrolyte (no additive): potential vs reversible capacity plots, respectively.

**
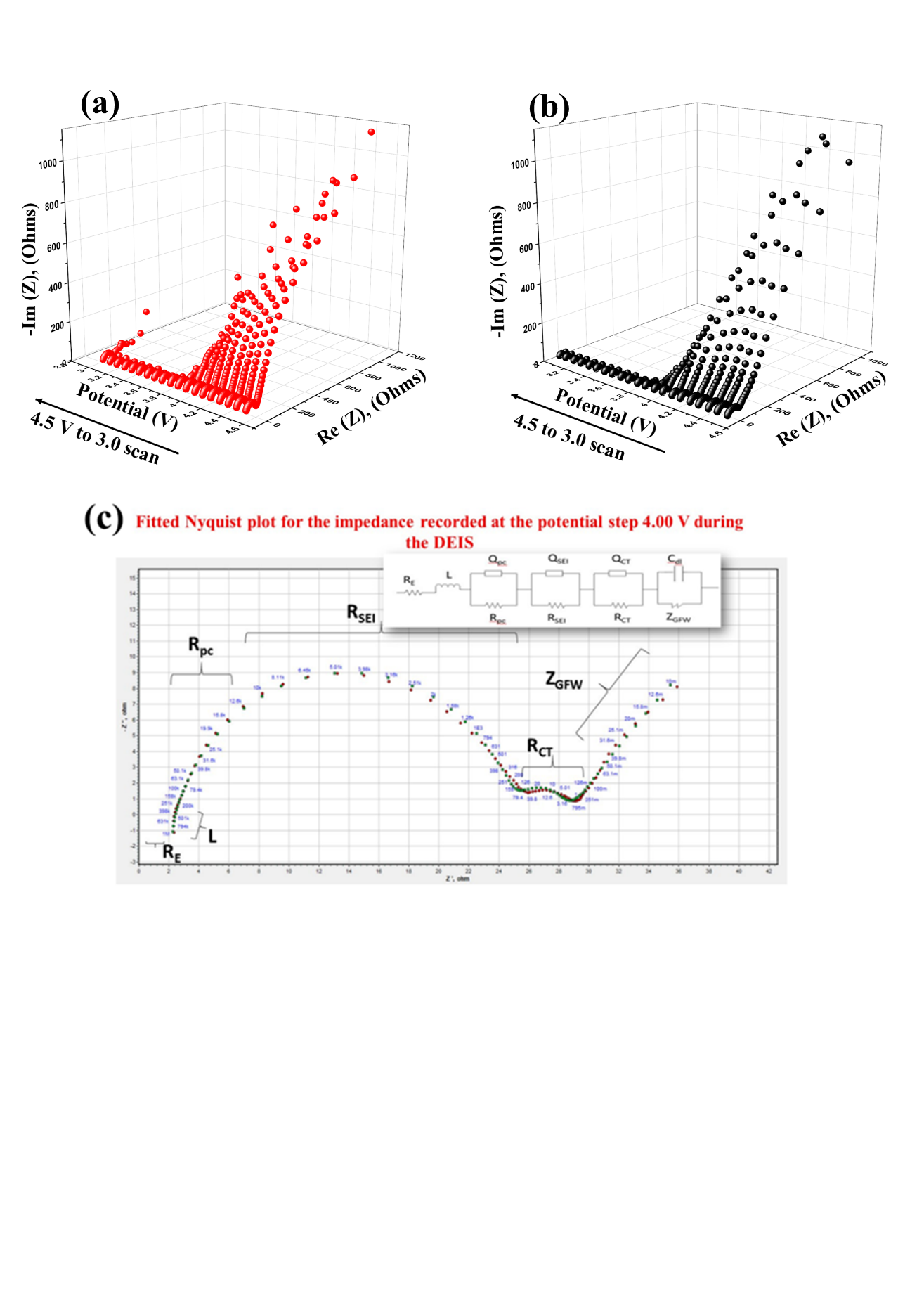
**

**Supplementary Figure S5:** DEIS 3-D Nyquist profiles after lithiation half-cycle of (a) DMBAP-based cathodic half-cell and (b) control system-based cathodic half-cell. (c) EECM fit representative Nyquist impedance profile of DMBAP-based cathodic half-cell recorded at the potential step of 4.0 V vs Li/Li^+^ during DEIS studies.

**Supplementary Table S3:** DEIS circuit fitting parameters during lithiation half-cycle of DMBAP-based cathodic half-cell**.**

| **R_s­_** | **R_PC_** | **R_CEI_** | **R_CT_** | **χ^2^** | **Circuit** | **Potential (V)** |
| --- | --- | --- | --- | --- | --- | --- |
| 1.16 | 1.82 | 24.58 | 11.94 | 0.00044 | R(L)(QR)(QR)(QR)(CW) | 3.0 |
| 1.94 | 0.33 | 24.81 | 10.84 | 4.3E-05 | R(L)(QR)(QR)(QR)(RW) | 3.07 |
| 3.55 | 1.94 | 25.05 | 9.48 | 1.24E-05 | R(L)(QR)(QR)(QR)(CW) | 3.15 |
| 2.99 | 2.96 | 29.53 | 15.93 | 1.1E-05 | R(L)(QR)(QR)(QR)(CW) | 3.22 |
| 1.41 | 3.00 | 10.38 | 12.94 | 1.03E-05 | R(L)(QR)(QR)(QR)(CW) | 3.3 |
| 2.68 | 10.83 | 2.394 | 11.99 | 1.93E-05 | R(L)(QR)(QR)(QR)(CW) | 3.37 |
| 1.95 | 2.84 | 119 | 10.84 | 1.84E-05 | R(L)(QR)(QR)(QR)(CW) | 3.45 |
| 5.92 | 3.84 | 28.64 | 9.73 | 1.83E-05 | R(L)(QR)(QR)(QR)(CW) | 3.52 |
| 1.09 | 1.74 | 23.34 | 17.8 | 1.9E-05 | R(L)(QR)(QR)(QR)(CW) | 3.6 |
| 0.55 | 1.22 | 15.33 | 19.94 | 2.94E-05 | R(L)(QR)(QR)(QR)(CW) | 3.67 |
| 1.99 | 3.94 | 13.06 | 20.84 | 5.0E-05 | R(L)(QR)(QR)(QR)(CW) | 3.75 |
| 4.92 | 5.82 | 17.17 | 12.94 | 5.42E-05 | R(L)(QR)(QR)(QR)(CW) | 3.82 |
| 1.72 | 1.94 | 19.58 | 11.84 | 1.85E-05 | R(L)(QR)(QR)(QR)(CW) | 3.9 |
| 0.57 | 1.04 | 5.909 | 5.98 | 1.61E-05 | R(L)(QR)(QR)(QR)(CW) | 3.97 |
| 0.06 | 20.47 | 6.32 | 2.04 | 1.84E-05 | R(L)(QR)(QR)(QR)(CW) | 4.05 |
| 2.74 | 19.99 | 12.96 | 1.94 | 5.61E-05 | R(L)(QR)(QR)(QR)(CW) | 4.12 |
| 1.99 | 45.91 | 12.02 | 0.93 | 8.92E-05 | R(L)(QR)(QR)(QR)(CW) | 4.2 |
| 9.38 | 8.43 | 12.02 | 5.92 | 7.05E-05 | R(L)(QR)(QR)(QR)(CW) | 4.27 |
| 1.73 | 1.22 | 12,94 | 1.03 | 6.96E-05 | R(L)(QR)(QR)(QR)(CW) | 4.35 |
| 0.72 | 4.09 | 11.843 | 1.56 | 4.44E-05 | R(L)(QR)(QR)(QR)(CW) | 4.42 |
| 11.39 | 0.93 | 10.48 | 1.83 | 2.82E-05 | R(L)(QR)(QR)(QR)(CW) | 4.5 |

**Supplementary Table S4:** DEIS circuit fitting parameters during lithiation half-cycle of control system.

| **R_s­_** | **R_PC_** | **R_CEI_** | **R_CT_** | **χ^2^** | **Circuit** | **Potential (V)** |
| --- | --- | --- | --- | --- | --- | --- |
| 10.33 | 23.94 | 70.21 | 97.71 | 0.0001 | R(L)(QR)(QR)(QR)(CW) | 4.5 |
| 19.57 | 35.85 | 72.89 | 91.73 | 3.4×10^-5^ | R(L)(QR)(QR)(QR)(CW) | 4.42 |
| 18.83 | 44.54 | 75.84 | 137.48 | 2.1E-05 | R(L)(QR)(QR)(QR)(CW) | 4.35 |
| 18.84 | 21.83 | 76.15 | 130.82 | 5.75E-05 | R(L)(QR)(QR)(QR)(CW) | 4.27 |
| 19.04 | 18.73 | 78.4 | 158.03 | 8.92E-05 | R(L)(QR)(QR)(QR)(CW) | 4.2 |
| 17.32 | 17.27 | 80.36 | 166.82 | 6.82E-05 | R(L)(QR)(QR)(QR)(CW) | 4.12 |
| 17.48 | 15.83 | 75.1 | 173.92 | 8.93E-05 | R(L)(QR)(QR)(QR)(CW) | 4.05 |
| 5.83 | 12.84 | 72.97 | 175.02 | 9.0E-05 | R(L)(QR)(QR)(QR)(CW) | 3.97 |
| 9.84 | 10.83 | 70.74 | 99.03 | 1.2E-05 | R(L)(QR)(QR)(QR)(CW) | 3.9 |
| 10.83 | 16.82 | 68.53 | 228.04 | 3.3E-05 | R(L)(QR)(QR)(QR)(CW) | 3.82 |
| 11.83 | 14.72 | 67.94 | 229.06 | 4.9E-05 | R(L)(QR)(QR)(QR)(CW) | 3.75 |
| 12.84 | 83.74 | 65.79 | 55.28 | 3.91E-05 | R(L)(QR)(QR)(QR)(CW) | 3.67 |
| 16.83 | 55.81 | 63.69 | 59.01 | 7.83E-05 | R(L)(QR)(QR)(QR)(CW) | 3.6 |
| 17.84 | 83.70 | 63.57 | 67.02 | 8.83E-05 | R(L)(QR)(QR)(QR)(CW) | 3.52 |
| 18.44 | 27.17 | 62.47 | 63.95 | 9.95E-05 | R(L)(QR)(QR)(QR)(CW) | 3.45 |
| 12.94 | 28.62 | 62.19 | 74.72 | 6.32E-05 | R(L)(QR)(QR)(QR)(CW) | 3.37 |
| 19.73 | 12.73 | 62.59 | 154.86 | 5.92E-05 | R(L)(QR)(QR)(QR)(CW) | 3.3 |
| 10.9 | 74.65 | 60.01 | 144.65 | 4.72E-05 | R(L)(QR)(QR)(QR)(CW) | 3.22 |
| 1.88 | 58.61 | 66.49 | 115.72 | 5.55E-05 | R(L)(QR)(QR)(QR)(CW) | 3.15 |
| 0.83 | 37.51 | 61.04 | 17.51 | 1.04E-05 | R(L)(QR)(QR)(QR)(CW) | 3.07 |
| 5.82 | 40.14 | 69.44 | 88.43 | 1.1E-05 | R(L)(QR)(QR)(QR)(CW) | 3.0 |


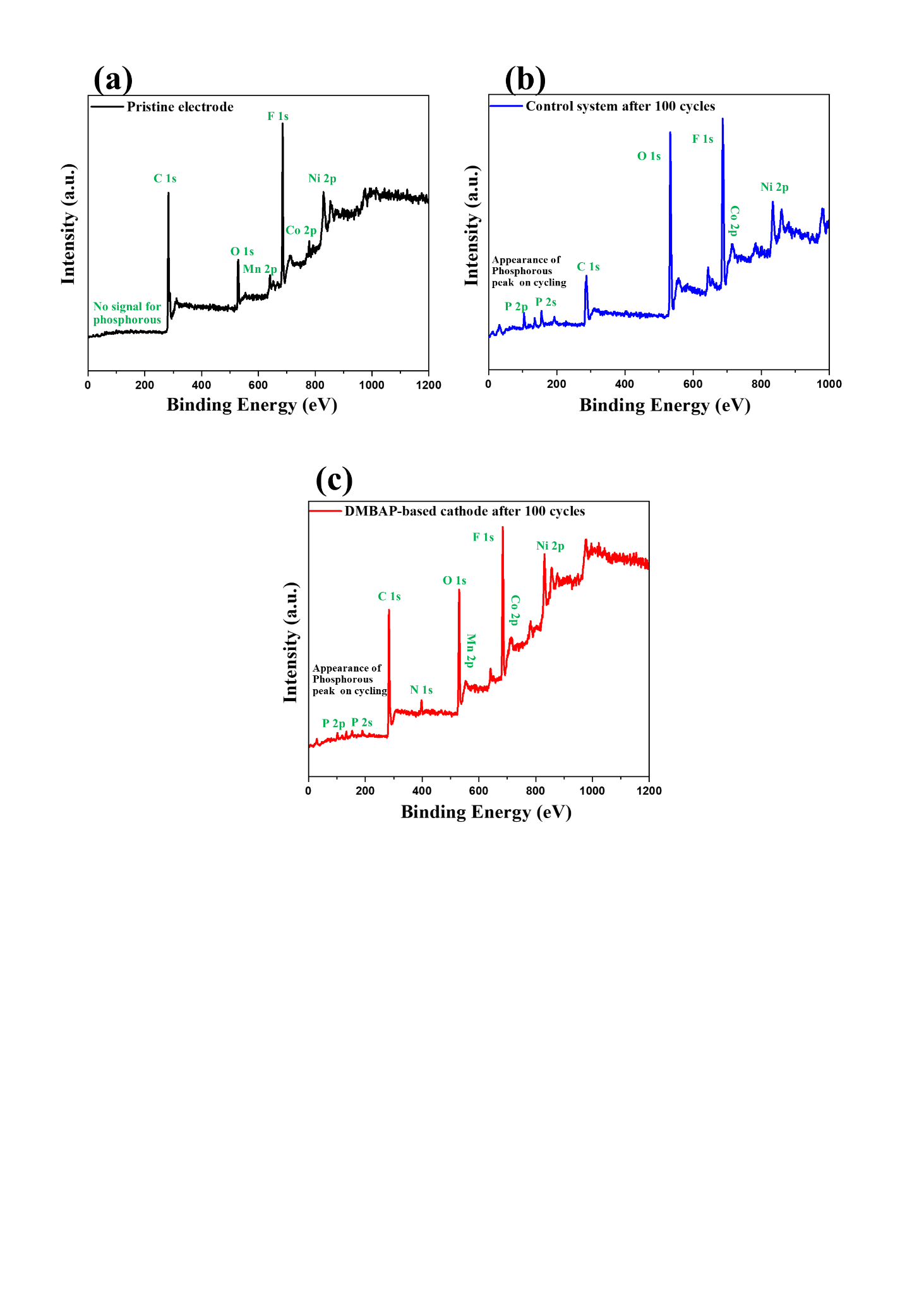


**Supplementary Figure S6:** XPS survey spectra of (a) Pristine electrode, (b) control system electrode after 100 cycles, and (c) DMBAP additive-based cathode after 100 cycles, respectively.


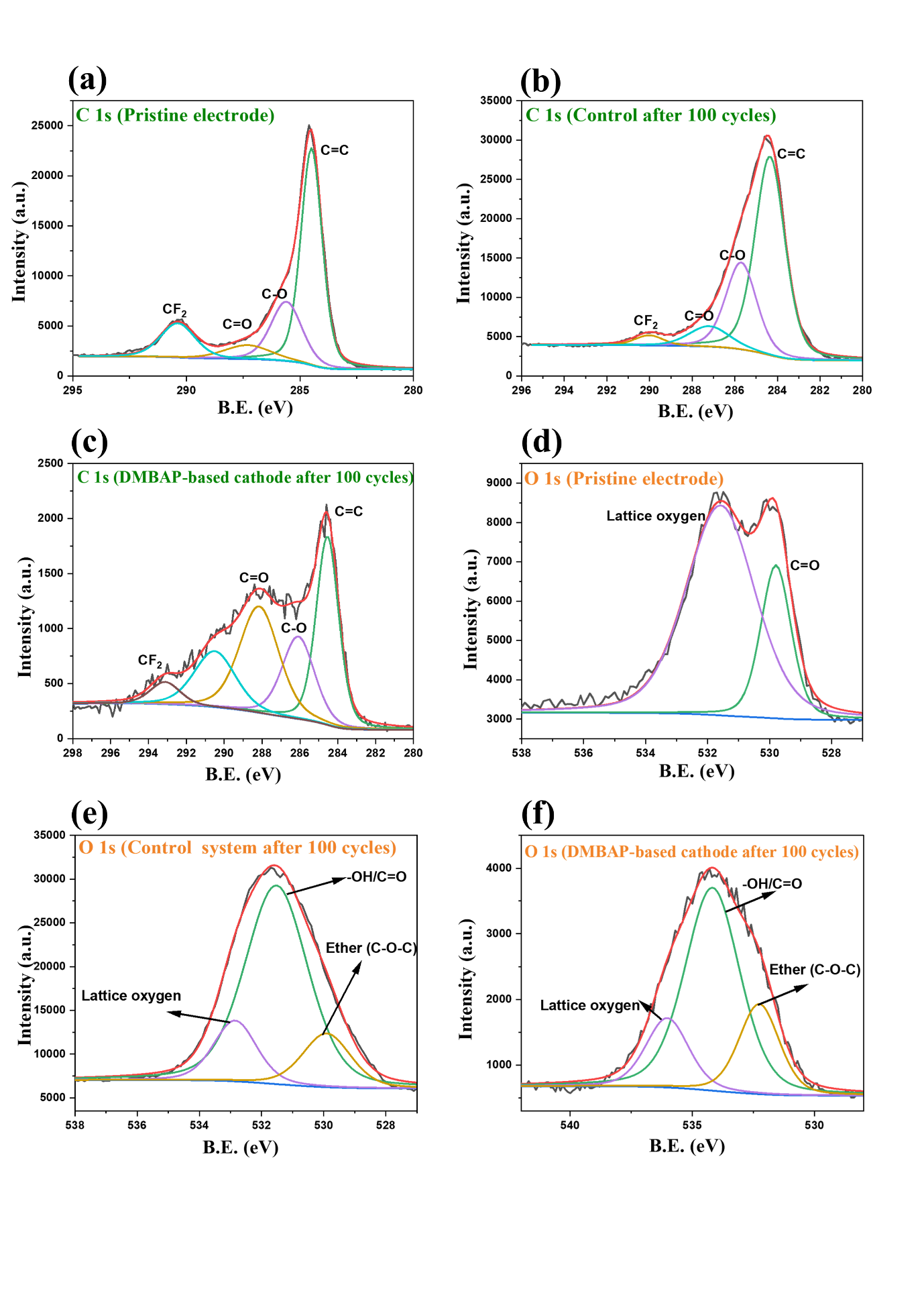


**Supplementary Figure S7:** XPS C 1s spectra of (a) Pristine electrode, (b) control system-based electrode after cycling, and (c) DMBAP-based electrode after cycling and O 1s spectra of (a) Pristine electrode, (b) control system-based electrode after cycling, and (c) DMBAP-based electrode after cycling, respectively.


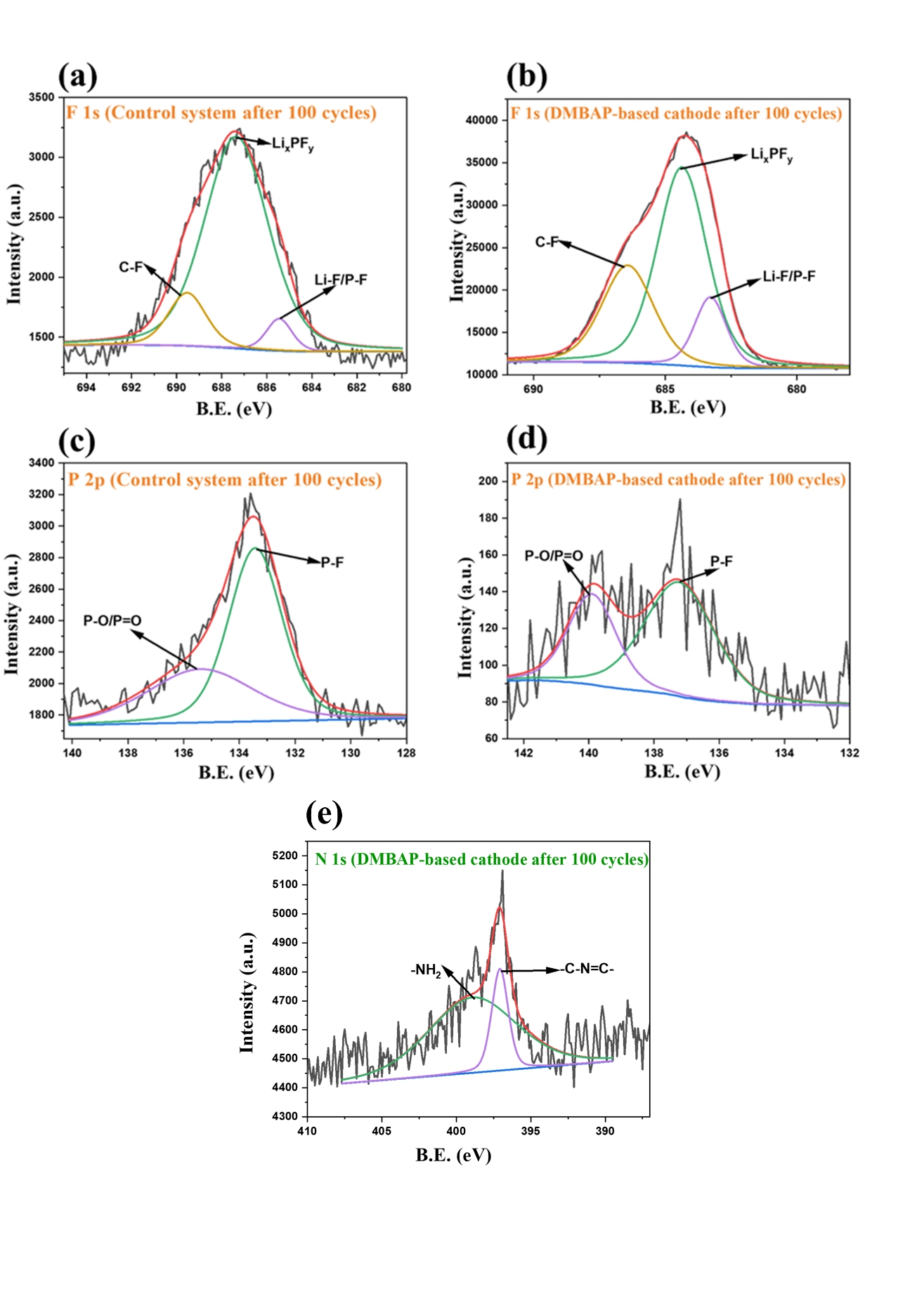


**Supplementary Figure S8:** XPS F 1s spectra of (a) control system (without additive)-based electrode and (b) DMBAP additive-based electrode. P 2p spectra of (a) control system (without additive)-based electrode and (b) DMBAP additive-based electrode, respectively. (e) N1 s spectrum of DMBAP additive-based electrode.
